# Influenza Vaccine Effectiveness During the 2023/2024 Season: A Test‐Negative Case–Control Study Among Emergency Hospital Admissions With Respiratory Conditions in Northern Ireland

**DOI:** 10.1111/irv.70149

**Published:** 2025-09-10

**Authors:** Magda Bucholc, Mark G. O'Doherty, Declan T. Bradley

**Affiliations:** ^1^ Public Health Agency Belfast UK; ^2^ Centre for Public Health Queen's University Belfast Belfast UK

**Keywords:** emergency admission, hospitalisation, influenza, influenza A, influenza B, vaccine, vaccine effectiveness

## Abstract

**Background:**

We evaluated the effectiveness of the influenza vaccine programme against infection among emergency hospital admissions with respiratory conditions in Northern Ireland during the 2023/2024 influenza season.

**Methods:**

Using a test‐negative design, we compared the odds of vaccination between patients who tested positive (cases) and negative (controls) for laboratory‐confirmed influenza, adjusting for confounders. VE was stratified by age group, sex and time since vaccination.

**Results:**

We included 2368 hospitalised patients, of whom 1740 (73.5%) were influenza positive. Among these, 1703 (97.9%) were influenza A and 37 (2.1%) were influenza B. Of the influenza A‐positive specimens, 84 were A(H1), 268 A(H3) and 1351 were untyped influenza A. VE against all laboratory‐confirmed influenza was 47.5% (95% CI: 31.3%–60.1%), including 65.2% (95% CI: 44.2%–78.6%) in children aged 2–17, 46% (95% CI: 7.8%–68.2%) in adults 18–64 and 39.5% (95% CI: 4.8%–62.1%) in adults aged 65 and over. VE against infection for influenza A was 45.8% (95% CI: 25.1%–61%) in all age groups, but 64.7% (95% CI: 42.6%–78.6%) among children aged 2–17, 43.9% (95% CI: 3.7%–67.1%) among adults aged 18–64 years old and 39.6% (95% CI: 5%–62.1%) in adults aged ≥ 65 years. Being vaccinated was associated with 44.2% (95% CI: −13.3%‐73.1%) and 37.9% (95% CI: 5.5%–59.5%) reduced odds of influenza A(H1) and A(H3)‐associated community‐acquired emergency admissions. VE against infection for influenza B was 87.2% (95% CI: 43.1%–98.3%). VE was highest within 2–8 weeks of vaccination at 67.5% (95% CI: 42.7%–81.7%) and declined to 41.2% (95% CI: 14.8%–59.5%) at 9–16 weeks.

**Conclusions:**

Influenza vaccines provided protection against influenza‐associated illness across age groups during the 2023/2024 influenza season.

## 1 Introduction

Influenza is a major contributor to severe illness, hospitalisations and mortality worldwide, particularly among vulnerable populations such as the elderly, infants, pregnant women and individuals with underlying medical conditions [1, 2, 3]. In Europe, seasonal influenza is estimated to infect up to 20% of the population annually, with the highest burden of severe outcomes among older adults [3]. Estimates from 28 EU countries suggested an average of approximately 27,600 influenza‐associated respiratory deaths each winter (range: 16,200–39,000 deaths) [4]. Around 88% of these deaths occurred in individuals aged 65 and over, with mortality rates in this age group roughly 35 times higher than in those under 65. In the United States, surveillance data from six influenza seasons (2010–2011 to 2015–2016), collected through FluSurv‐NET—a system covering approximately 9% of the US population—showed that although older adults (≥ 65 years) had the lowest median influenza incidence (3.9%) compared to children (9.3%) and adults aged 18–64 years (8.9%), they accounted for 50%–70% of influenza‐related hospitalisations and 70%–85% of related deaths [5]. Despite representing only 1%–2% of medically attended cases, influenza‐related hospitalisations accounted for 70% of direct medical costs [6].

Recent evaluations of influenza vaccine effectiveness (VE) during the 2023/2024 season provide updated insights into vaccine performance across populations. Whitaker et al. [7], using data from primary care and hospital settings in the United Kingdom, reported VE estimates of 63%–65% in children aged 2–17, 36%–55% in adults aged 18–64 and 40%–55% in those ≥ 65, with slightly higher estimates observed in primary care settings. VE point estimates against influenza A(H3N2) were generally lower than those against influenza A(H1N1)pdm09. Their results are consistent with those reported for Canada during the early 2023/2024 season, which showed moderate VE during an influenza A(H1N1)pdm09‐dominated period [8]. In the United States, Tenforde et al. [9] analysed over 340,000 healthcare encounters across eight states and found VE of 58% against both hospitalisations and emergency/urgent care visits in children aged 6 months to 17 years and 39% and 47% effectiveness, respectively, in adults. VE was higher against influenza B than A. Similarly, Martínez‐Baz et al. [10] reported moderate VE in Spain, with 43% effectiveness against hospitalisation overall—61% in individuals < 65 years and 35% in those ≥ 65. VE was 48% against A/H1N1 but low (15%) against A/H3N2, with outpatient VE estimated at 49% overall.

In Northern Ireland (NI), during the 2023/2024 influenza season, indicators of influenza activity suggested low levels of circulation prior to December 2023 [11]. Influenza activity steadily increased throughout December, peaking in the last week of January 2024, before declining in early February. In mid‐February, influenza activity saw a slight increase, primarily driven by a rise in influenza B cases, before eventually returning to pre‐December 2023 levels by mid‐April 2024. During the 2023/2024 influenza season, influenza A was the dominant type, with A(H3) being the most prevalent subtype. Influenza type B circulated at low levels becoming more prominent in 2024 as influenza A declined [11].

The seasonal influenza vaccination campaign in NI began on 18 September 2023 and was coordinated across multiple distribution channels, including general practices, community pharmacies, Health and Social Care (HSC) Trusts and Trust school nursing teams. Eligibility for vaccination followed national guidance, prioritising individuals at higher risk of severe disease, including children aged 2–17 years, adults aged 65 years and older (and from January 2024, those aged 50–64), individuals aged 18–64 in clinical risk groups or at increased risk of severe influenza‐related outcomes, children in clinical risk groups aged 6 months to less than 2 years, pregnant women, carers and close contacts aged 18–64 and frontline HSC workers [12]. In line with Joint Committee on Vaccination and Immunisation (JCVI) recommendations, specific influenza vaccines were allocated to eligible groups during the 2023/2024 season [12]. Individuals aged 65 years and over were primarily offered the adjuvanted quadrivalent inactivated influenza vaccine (aQIV). The quadrivalent cell‐based influenza vaccine (QIVc) was recommended for adults aged 18–64 years in clinical risk groups, including pregnant women, as well as for carers, close contacts of immunocompromised individuals and frontline HSC workers. For children aged 2 years to under 18 years, the live attenuated influenza vaccine (LAIV) was used, except where contraindicated. In such cases, QIVc was offered as an alternative. QIVc was also administered to children in clinical risk groups aged 6 months to under 2 years. The total number of vaccinations administered during the 2023/2024 programme was 442,563. Influenza vaccine uptake in eligible adults was highest in care home residents (82%) and those aged 65 years and over (78%). Uptake of influenza vaccine in eligible children was highest in primary school children at 69%. This study aims to evaluate the effectiveness of the influenza vaccine programme against infection among emergency hospital admissions with respiratory conditions in NI during the 2023/2024 influenza season.

## 2 Methods

### 2.1 Study Design

We conducted a test‐negative case–control study to estimate end‐of‐season VE against laboratory‐confirmed influenza during the 2023/2024 influenza season in NI. The test‐negative design is widely used in influenza VE studies and helps minimise bias by comparing vaccination status between patients who test positive (cases) and those who test negative (controls) for influenza, all of whom sought care and were tested for similar respiratory symptoms. Cases were defined as patients admitted to hospital through emergency care who had a positive influenza result from a reverse‐transcription polymerase chain reaction (RT‐PCR) test. Controls were patients admitted via emergency care who tested negative for influenza but had a laboratory‐confirmed alternative respiratory pathogen, including respiratory syncytial virus (RSV), metapneumovirus, rhinovirus, adenovirus, parainfluenza, pneumocystis, enterovirus, *Bordetella pertussis* , *Bordetella parapertussis* , *Mycoplasma pneumoniae* , *Haemophilus influenzae* , *Streptococcus pneumoniae* , parvovirus or measles. This strict control definition was used to minimise potential bias from patients tested for non‐respiratory reasons or with low clinical suspicion of influenza. Individuals who tested positive for SARS‐CoV‐2 were excluded from the control group.

### 2.2 Study Period

The study period spanned from ISO Week 40 of 2023 (beginning 2 October 2023) through ISO Week 17 of 2024 (ending 28 April 2024), corresponding with the national influenza vaccine rollout.

### 2.3 Data Sources

Vaccination status, influenza test results and hospital admissions data were obtained through deterministic linkage of individual‐level data from three sources: the NI Vaccine Management System (VMS), the regional influenza surveillance system and administrative hospital admissions records from HSC information systems. Linkage was carried out by the authors using a pseudonymised national health identifier (HSC number).

The VMS serves as the official system of record for six key vaccination programmes (influenza, RSV, shingles, pertussis, Covid‐19 and MMR) administered across NI. The system supports the delivery of vaccines to those individuals who are eligible for vaccination under the UK immunisation programme including both routine and seasonal campaigns. Development of the VMS commenced in 2020. Initially, the system was jointly controlled by the Health and Social Care Board (HSCB) and the Public Health Agency (PHA). From 2022, operational responsibility shifted jointly to PHA and the Department of Health (DoH) via the Strategic Planning and Performance Group (SPPG) following the closure of HSCB. In 2023, PHA took on single control ownership of the VMS. The system captures data from all eligible individuals vaccinated through general practices, community pharmacies and school‐based programmes. Influenza vaccinations delivered through the school‐based programmes are initially recorded in the NI Child Health System (CHS), which feeds into the VMS.

Influenza testing data were sourced from the regional influenza surveillance system, which collates virological reports from the Regional Virus Laboratory (RVL) and all local HSC Trust laboratories.

Hospital admission data were obtained from the Patient Administration System (PAS) and the Epic electronic health record system, the latter introduced in the South Eastern HSC Trust on 6 November 2023 and in the Belfast HSC Trust on 6 June 2024. NI has six HSC Trusts in total—Belfast (BHSCT), South Eastern (SEHSCT), Southern (SHSCT), Northern (NHSCT), Western (WHSCT) and NI Ambulance Service—although only five of these (BHSCT, SEHSCT, SHSCT, NHSCT and WHSCT) provide acute hospital services. All acute hospitals within the five acute‐service Trusts contributed data to this study through their respective systems. In total, data from 10 acute hospitals were included. Following the implementation of Epic, consistent identification of emergency admissions was not yet possible; therefore, from the respective go‐live dates, all community‐acquired admissions were included. Prior to these dates, only admissions explicitly recorded as ‘emergency’ were considered.

All data sources—VMS, laboratory testing data and hospital admissions—were linked and analysed within the Northern Ireland Health Analytics Platform (NIHAP). NIHAP is maintained by Digital Health and Care Northern Ireland (DHCNI) and was established in 2020 to support the PHA pandemic response. It is hosted within Microsoft Azure Synapse Analytics, under the Belfast HSC secure cloud tenancy, and continues to serve as a key surveillance platform post‐pandemic.

### 2.4 Study Population and Eligibility Criteria

The study population included all individuals aged 2 years and older who had an emergency hospital admission and a recorded influenza RT‐PCR test during the study period. Community‐acquired influenza admissions were defined as emergency admissions in which a positive influenza test was obtained up to 7 days before or within 1 day following the date of admission. It was assumed that patients tested within this window had at least one acute respiratory infection (ARI) symptom at the time of testing. Only the first admission per infection episode was included. Influenza infection episodes were defined using a 42‐day (6‐week) rolling window from the date of the first positive test result; any subsequent positive specimens for the same individual within this period were considered part of the same episode, whereas positive specimens occurring more than 42 days after the last specimen were classified as new episodes. Patients were considered vaccinated if they had received a dose of the seasonal influenza vaccine at least 14 days prior to their sample date. Those with no record of influenza vaccination before the sample date during the 2023/2024 campaign were considered unvaccinated. Patients who tested positive for influenza within 14 days of receiving the vaccine were excluded from the main analysis (*n* = 45) to allow for the time required for an adequate immune response to develop.

### 2.5 Laboratory Testing

Respiratory samples were tested using RT‐PCR. Influenza A viruses were subtyped as either A(H1) or A(H3); neuraminidase subtyping was not conducted, so full subtype characterisation (e.g., A(H1N1)) was unavailable. Lineage differentiation for influenza B viruses was also not performed.

### 2.6 Exposure and Outcome Definitions

The primary exposure was receipt of the 2023/2024 seasonal influenza vaccine, categorised as vaccinated (≥ 14 days before testing) or unvaccinated. The primary outcome was laboratory‐confirmed influenza, defined as a positive RT‐PCR result for influenza A or B.

### 2.7 Statistical Analysis

Comparisons of continuous variables were performed using the Wilcoxon rank‐sum test, given the non‐normal distribution of the data. Adjusted odds ratios (aORs) for influenza vaccination among cases and controls were estimated using multivariable logistic regression. Covariates considered for inclusion as potential confounders were age, sex, month of test and HSC Trust (BHSCT, SEHSCT, SHSCT, NHSCT and WHSCT) identified through a pre‐specified variable selection approach based on epidemiological relevance. VE was then calculated as (1 − aOR) × 100. The assumption of linearity between age and outcome was tested by comparing models with age specified as a linear term, a spline term and a generalised additive model. The likelihood ratio test indicated that the linearity assumption did not hold (*p* < 0.001), and therefore, age was modelled using 16 narrow age categories: 2–9, 10–17, 18–24, 25–29, 30–34, 35–39, 40–44, 45–49, 50–54, 55–59, 60–64, 65–69, 70–74, 75–79, 80–84 and ≥ 85 years.

VE was estimated for any influenza infection and separately for influenza A and B. Further stratified analyses were conducted by predefined age groups (2–17, 18–64, 65+), sex and time since vaccination. To evaluate whether VE differed significantly across predefined subgroups, we conducted additional analyses including an interaction term between vaccination status and the stratifying variable (e.g., sex) in the logistic regression model. Likelihood ratio tests were used to compare the interaction model to the main effects model. These interaction models were used solely to assess statistical evidence of effect modification and are not presented in the main results. All analyses were performed using R Version 4.4.0.

### 2.8 Sensitivity Analyses

Four sensitivity analyses were conducted to assess the robustness of the primary findings. First, we included individuals classified as partially vaccinated—those who received the influenza vaccine 7–14 days prior to their sample date—to explore the potential for early protection following vaccination. Second, we included SARS‐CoV‐2‐positive individuals as controls to evaluate whether their exclusion in the primary analysis introduced selection bias [13]. Third, we present VE estimates from models in which age was included as a restricted cubic spline. This alternative modelling approach was undertaken because the assumption of a linear relationship between age and the outcome was not satisfied (*p* < 0.001). Spline modelling allowed for a more flexible characterisation of age as a continuous variable, with knot placement determined separately for different sub‐analyses to best capture non‐linear associations. Finally, to address potential bias in subgroups with small numbers of cases, such as influenza B and influenza A(H1), we re‐estimated VE using Firth's penalised logistic regression. This approach reduces small‐sample bias by penalising maximum likelihood estimates.

## 3 Results

### 3.1 Study Population

**FIGURE 1:**
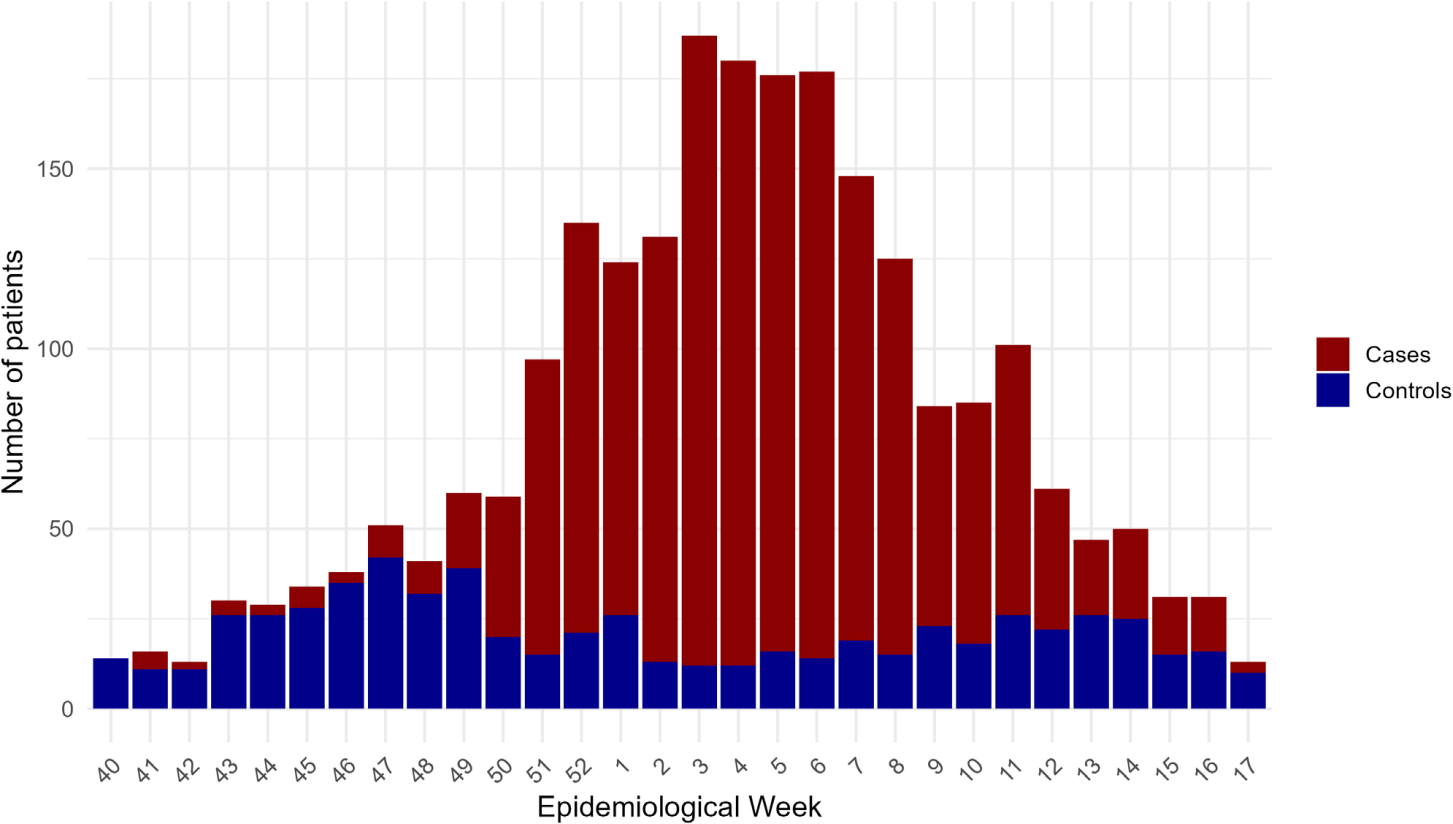
Weekly number of influenza cases and test‐negative controls included in the study. Data are shown by epidemiological week of sample collection from Week 40 of 2023 to Week 17 of 2024.

We included 2368 hospitalised patients, of whom 1740 (73.5%) were influenza positive (Table 1). Among these, 1703 (97.9%) were influenza A, and 37 (2.1%) were influenza B. Of the influenza A‐positive specimens, 84 were A(H1), 268 A(H3) and 1351 were untyped influenza A. Among controls (those that tested negative for influenza and were admitted to hospital with a result indicating another respiratory infection), the proportion with the influenza vaccine was 42.3% (*n* = 266) compared to 41.2% (*n* = 717) among all influenza cases, 42% (*n* = 7152) among influenza A cases and 5.4% (*n* = 2) among influenza B cases. The median age was 62 years among all influenza cases. There were differences between the influenza A and B cases in terms of age (median = 62 vs. 27, respectively; *p* < 0.001). The weekly number of included influenza cases and controls across the study period is shown in Figure 1.

**TABLE 1:** Characteristics of cases and controls included in the study by vaccine status, 2023/2024 influenza season in Northern Ireland. The five HSC Trusts included in the analysis were: Belfast (BHSCT), South Eastern (SEHSCT), Southern (SHSCT), Northern (NHSCT) and Western (WHSCT).

	Controls		Cases		Overall	
	Unvaccinated (*N* = 362)	Vaccinated (N = 266)	Unvaccinated (*N* = 1023)	Vaccinated (N = 717)	Unvaccinated (*N* = 1385)	Vaccinated (*N* = 983)
Age						
2–17	233 (64.4%)	107 (40.2%)	247 (24.1%)	85 (11.9%)	480 (34.7%)	192 (19.5%)
18–64	74 (20.4%)	42 (15.8%)	473 (46.2%)	136 (19.0%)	547 (39.5%)	178 (18.1%)
65+	55 (15.2%)	117 (44.0%)	303 (29.6%)	496 (69.2%)	358 (25.8%)	613 (62.4%)
Sex						
Female	190 (52.5%)	164 (61.7%)	581 (56.8%)	365 (50.9%)	771 (55.7%)	529 (53.8%)
Male	172 (47.5%)	102 (38.3%)	442 (43.2%)	352 (49.1%)	614 (44.3%)	454 (46.2%)
Month of test						
October	55 (15.2%)	11 (4.1%)	9 (0.9%)	3 (0.4%)	64 (4.6%)	14 (1.4%)
November	81 (22.4%)	61 (22.9%)	17 (1.7%)	9 (1.3%)	98 (7.1%)	70 (7.1%)
December	65 (18.0%)	47 (17.7%)	166 (16.2%)	93 (13.0%)	231 (16.7%)	140 (14.2%)
January	42 (11.6%)	31 (11.7%)	376 (36.8%)	255 (35.6%)	418 (30.2%)	286 (29.1%)
February	35 (9.7%)	32 (12.0%)	291 (28.4%)	241 (33.6%)	326 (23.5%)	273 (27.8%)
March	51 (14.1%)	51 (19.2%)	126 (12.3%)	95 (13.2%)	177 (12.8%)	146 (14.9%)
Missing	33 (9.1%)	33 (12.4%)	38 (3.7%)	21 (2.9%)	71 (5.1%)	54 (5.5%)
HSC Trust						
BHSCT	21 (5.8%)	31 (11.7%)	197 (19.3%)	120 (16.7%)	218 (15.7%)	151 (15.4%)
NHSCT	27 (7.5%)	22 (8.3%)	274 (26.8%)	224 (31.2%)	301 (21.7%)	246 (25.0%)
SEHSCT	51 (14.1%)	57 (21.4%)	207 (20.2%)	132 (18.4%)	258 (18.6%)	189 (19.2%)
SHSCT	135 (37.3%)	82 (30.8%)	211 (20.6%)	143 (19.9%)	346 (25.0%)	225 (22.9%)
WHSCT	127 (35.1%)	74 (27.8%)	128 (12.5%)	97 (13.5%)	255 (18.4%)	171 (17.4%)
Unknown	1 (0.3%)	0 (0%)	6 (0.6%)	1 (0.1%)	7 (0.5%)	1 (0.1%)

Vaccine types varied by age group: Children aged 2–17 primarily received the LAIV via nasal spray, adults aged 18–64 years mostly received the quadrivalent cell‐based vaccine (QIVc), and adults aged ≥ 65 years mostly received the adjuvanted egg‐based vaccine (aQIV). Among controls, the proportions of each vaccine type administered within each age group were as follows: In those aged 2–17 years, 93.5% received LAIV and 6.5% received QIVc; in those aged 18–64 years, 97.6% received QIVc and 2.4% received aQIV; and in those aged ≥ 65 years, 99.1% received aQIV and 0.9% received QIVc.

### 3.2 VE

**FIGURE 2:**
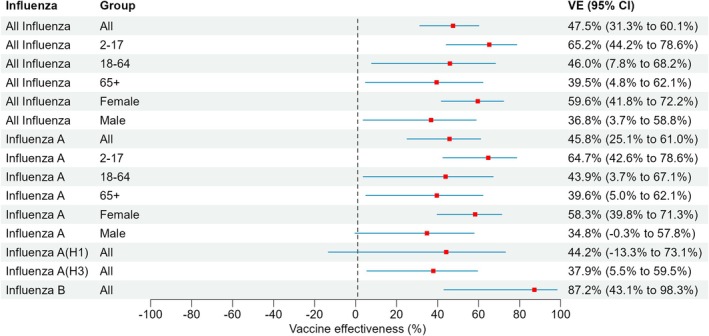
Influenza vaccine effectiveness in preventing community‐acquired emergency hospitalisations due to laboratory‐confirmed influenza overall and by age group, sex and influenza type/subtype, 2023/2024 influenza season in Northern Ireland. VE estimates adjusted for age group (2–9, 10–17, 18–24, 25–29, 30–34, 35–39, 40–44, 45–49, 50–54, 55–59, 60–64, 65–69, 70–74, 75–79, 80–84 and ≥ 85 years), sex, month of test and HSC Trust. CI, confidence interval.

VE for all studies is summarised in Figure 2. Sample size did not permit estimation of VE against influenza A(H1), A(H3) and B stratified by age group and sex. VE against any influenza among all ages was 47.5% (95% CI: 31.3%–60.1%). VE was 65.2% (95% CI: 44.2%–78.6%) for children aged 2–17 years, 46.0% (95% CI: 7.8%–68.2%) for adults aged 18–64 years and 39.5% (95% CI: 4.8%–62.1%) for adults aged ≥ 65 years. Among females, VE was 59.6% (95% CI: 41.8%–72.2%) although it was 36.8% (95% CI: 3.7%–58.8%) among males (*p* = 0.007).

VE against infection for influenza A virus type was estimated to be 45.8% (95% CI: 25.1%–61.0%) among all age groups, but 64.7% (95% CI: 42.6%–78.6%) among children aged 2–17, 43.9% (95% CI: 3.7%–67.1%) among adults aged 18–64 years old and 39.6% (95% CI: 5.0%–62.1%) among adults aged ≥ 65 years. The vaccine was 44.2% (95% CI: −13.3%–73.1%) and 37.9% (95% CI: 5.5%–59.5%) effective at preventing influenza A(H1)‐ and A(H3)‐associated community‐acquired emergency admissions. For influenza B, overall VE was 87.2% (95% CI: 43.1%–98.3%).

**FIGURE 3:**
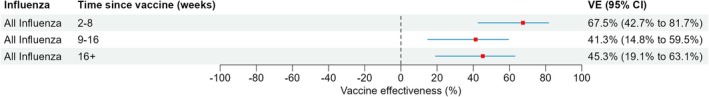
Influenza vaccine effectiveness by time since vaccination in weeks. VE estimates adjusted for age group (2–9, 10–17, 18–24, 25–29, 30–34, 35–39, 40–44, 45–49, 50–54, 55–59, 60–64, 65–69, 70–74, 75–79, 80–84 and ≥ 85 years), sex, month of test and HSC Trust. CI, confidence interval.

To evaluate the duration of protection offered by influenza vaccination, we estimated VE by time since vaccination, grouped into intervals of 2–8 weeks, 9–16 weeks and ≥ 16 weeks post‐vaccination (Figure 3). VE was highest within 2–8 weeks of vaccination at 67.5% and declined to 41.2% at 9–16 weeks and 45.3% at ≥ 16 weeks. These findings suggest waning of vaccine‐induced immunity over the course of the influenza season but continued benefit beyond 4 months.

### 3.3 Sensitivity Analyses

We conducted four sensitivity analyses to assess the robustness of the VE estimates. First, we included individuals who were tested 7–14 days after vaccination to explore the impact of partial vaccination under the assumption that some immune response may already have begun to develop. VE estimates in this analysis were generally similar to, or slightly higher than, those in the main analysis (Figure S1), potentially reflecting the early onset of immune protection during this period. Second, to evaluate potential selection bias, we included individuals with a positive SARS‐CoV‐2 test in the control group. VE estimates differed by no more than 2.3 percentage points compared to the main analysis (Figure S2), suggesting this had minimal influence on the results. Third, we explored the effect of different age modelling approaches by re‐estimating VE using restricted cubic splines instead of categorical age groups. Overall, the results were closely aligned with those from the primary analysis, with slightly higher VE for influenza A and B overall, though some variation was observed in age‐ and sex‐stratified estimates (Figure S3). This indicates that while the method of age modelling had limited impact overall, model flexibility may influence subgroup estimates. Finally, we applied Firth's penalised logistic regression to account for potential bias arising from small sample sizes in certain subgroups, particularly for influenza A(H1), A(H3) and B. This method yielded slightly lower VE estimates than standard logistic regression (Figure S4), likely due to the shrinkage of extreme estimates towards the null in the presence of sparse data.

## 4 Discussion

This study assessed the effectiveness of the 2023/2024 seasonal influenza vaccine among emergency hospital admissions for respiratory conditions in NI. Among the 2368 patients included, the vaccine was found to provide protection against both influenza A and B. These results are consistent with findings from other countries, including the United Kingdom, Canada and Spain, which also reported moderate VE during the 2023/2024 season [7, 8, 10].

Compared with the 2022/2023 season, VE estimates were generally higher in our study. In the previous season, data from six European studies covering 16 countries reported VE against influenza A ranging from 27% to 44% across all ages, with the lowest estimates seen in adults aged ≥ 65 years, and from 64% to 85% for influenza B [14]. The higher VE observed in our study may reflect the benefit of updating the A(H1N1)pdm09 component of the northern hemisphere vaccine for 2023/2024.

Our overall VE estimate of 47.5% aligns with mid‐ and end‐of‐season estimates from Europe and the United States, which ranged from 38% to 44% [7, 9]. Effectiveness was higher in individuals aged < 65 years—65.2% in children aged 2–17 years and 46% in adults aged 18–64—compared with 39.5% in those aged ≥ 65 years. Although confidence intervals overlapped between age groups, VE point estimates were consistently higher in children across primary and sensitivity analyses. This age‐specific pattern in VE may reflect the routine use of LAIV in the UK's childhood immunisation programme, which appears to have performed well during the 2023/2024 season.

The high VE observed in children is consistent with findings from the UK primary care study (GB‐PC) and hospital‐based studies in England (EN‐H) and Scotland (SC‐H), all of which reported VE estimates between 63% and 65% for this age group [7]. Similarly, our VE estimates in adults aged 18–64 and those ≥ 65 years are in line with the 2023/2024 UK‐based studies which reported VE ranging from 36% to 55% in adults aged 18–64 and 40% to 55% in those aged ≥ 65 years [7]. In the United States, Tenforde et al. [9] reported VE of 58% against both hospitalisations and emergency/urgent care visits in children aged 6 months to 17 years. Among adults, VE ranged from 39% to 47%, reflecting the expected age‐related decline in vaccine‐induced immunity [9]. Across studies, VE against influenza B was generally higher than for influenza A subtypes [15, 16].

The lower VE against influenza A(H3) observed in our study is consistent with prior evidence showing reduced effectiveness for this subtype compared with A(H1). For example, in Spain, Martínez‐Baz et al. [10] reported VE of 48% against A(H1N1)pdm09 and only 15% against A(H3N2), although the number of A(H3N2) cases was limited. In the US, Tenforde et al. [9] reported VE of 54%–61% against A(H1N1)pdm09 in outpatient settings and 60% against hospitalisations, whereas VE against A(H3N2) was lower at around 55%. In Canada, VE reached 61% for A(H1N1) and 49% for A(H3N2) [8].

VE against influenza B was particularly high in our study, at 87.2%, exceeding estimates from several other sources [15, 16]. Chung et al. [15] reported VE of 74% against B/Victoria. The Centers for Disease Control and Prevention data from 2023/2024 showed VE against influenza B ranging from 64% to 89% in children in outpatient settings and from 60% to 78% for all adults across settings [16]. VE against influenza B‐associated hospitalisation was estimated at 60% for all adults aged ≥ 18 years [16].

In the 2023/2024 season, VE against influenza B was consistently higher than that for A(H1) and A(H3), which likely reflects the slower mutation rate of influenza B viruses. Because influenza B evolves more slowly than influenza A, particularly A(H3), the selected vaccine strains are more likely to match circulating strains, resulting in greater protection [17]. In contrast, the rapid antigenic drift seen in A(H3) viruses often undermines vaccine match and reduces effectiveness.

This study has several strengths, including multiple sensitivity analyses and the use of a comprehensive, individual‐level data linkage across regional health systems in NI. The VMS, which includes data from general practices, community pharmacies and school‐based programmes, provides near‐complete coverage of influenza vaccinations administered to eligible individuals. Influenza laboratory data from the RVL and all HSC Trust laboratories ensure full regional surveillance, with all data securely linked and analysed within the NIHAP. However, certain limitations remain. A short lag between vaccine administration and data entry to VMS may introduce minor timing discrepancies. Additionally, eligibility categories recorded in VMS are not mutually exclusive, and individuals may qualify under multiple criteria, but only one reason is typically recorded—this may affect uptake estimates by risk group. This does not however affect vaccination status ascertainment or our VE results that were not adjusted by the eligibility group. Furthermore, patient age was determined based on the difference between date of birth and the date of sample collection, rather than the date of vaccination. As a result, the reported distibution of vaccine types across age categories may be slightly misrepresented. In hospitals within SEHSCT and BHSCT using the newer Epic system, emergency admission coding was not yet available, leading to the inclusion of all community‐acquired admissions (‘emergency and other’) from Epic's go‐live dates. However, during the preceding season, 95.7% of community‐acquired admissions in SEHSCT and 89.1% in BHSCT were emergency admissions, suggesting the potential for misclassification is low. Finally, small sample sizes in some subgroups limited VE estimation by age or virus subtype, and the possibility of residual confounding from unmeasured factors, such as underlying health conditions or previous vaccination history, cannot be excluded.

## 5 Conclusion

Taken together, the 2023/2024 VE estimates in NI are encouraging and demonstrate substantial protection against hospitalisation due to both influenza A and B. In our analysis, fewer than half of the test‐negative control patients had received the influenza vaccine. The overall public health benefit of annual influenza vaccination depends not only on VE but also on the level of vaccination coverage. Increasing uptake is essential to maximise the prevention of influenza‐associated illness and reduce related hospitalisation [18, 19]. Overall, our findings reinforce the value of the seasonal influenza immunisation programme in NI in preventing emergency hospital admissions due to influenza.

## Author Contributions

**Magda Bucholc:** investigation, methodology, validation, formal analysis, writing – original draft. **Mark G. O'Doherty:** data curation, writing – review and editing, validation, investigation. **Declan T. Bradley:** conceptualisation, methodology, investigation, validation, writing – review and editing.

## Ethics Statement

Public Health Agency has permission to process patient confidential information for national surveillance of communicable diseases under the Health and Social Care Act (Northern Ireland) 2016. As such, specific ethical approval was not necessary.

## Conflicts of Interest

The authors declare no conflicts of interest.

## Peer Review

The peer review history for this article is available at https://www.webofscience.com/api/gateway/wos/peer‐review/10.1111/irv.70149.

## Supporting information


**Figure S1:** Vaccine effectiveness estimates including partially vaccinated individuals (sample collected 7–14 days post vaccination). VE adjusted for age group (2–9, 10–17, 18–24, 25–29, 30–34, 35–39, 40–44, 45–49, 50–54, 55–59, 60–64, 65–69, 70–74, 75–79, 80–84 and ≥ 85 years), sex, month of test and trust area. CI, confidence interval.
**Figure S2:** Vaccine effectiveness estimates including controls who tested positive for SARS‐CoV‐2. VE adjusted for age group (2–9, 10–17, 18–24, 25–29, 30–34, 35–39, 40–44, 45–49, 50–54, 55–59, 60–64, 65–69, 70–74, 75–79, 80–84 and ≥ 85 years), sex, month of test and HSC Trust. CI, confidence interval.
**Figure S3:** Vaccine effectiveness estimates against all influenza and by influenza type/subtype, age group and sex. All odds ratios were adjusted for sex, month of test, HSC Trust and age, parametrised as a restricted cubic spline, with k‐knots determined separately for different sub‐analyses. CI, confidence interval.
**Figure S4:** Vaccine effectiveness estimates against influenza B, A(H1) and A(H3) based on Firth's penalised logistic regression models. VE adjusted for age group (2–9, 10–17, 18–24, 25–29, 30–34, 35–39, 40–44, 45–49, 50–54, 55–59, 60–64, 65–69, 70–74, 75–79, 80–84 and ≥ 85 years), sex, month of test and HSC Trust. Error bars represent 95% confidence intervals (CI).

## Data Availability

Data supporting this study cannot be made available due to ethical and legal reasons.
