# Supplementary material for: Influenza Vaccine Effectiveness During the 2023/2024 Season: A Test‐Negative Case–Control Study Among Emergency Hospital Admissions With Respiratory Conditions in Northern Ireland
Source: Influenza Other Respir Viruses. 2025 Sep 10;19(9):e70149. doi: 10.1111/irv.70149 (PMC12422802; doi:10.1111/irv.70149)

**Supplementary material**

**Figure S1.** Vaccine effectiveness estimates including partially vaccinated individuals (sample collected 7-14 days post vaccination). VE adjusted for age group (2–9, 10-17, 18-24, 25-29, 30-34, 35-39, 40-44, 45-49, 50-54, 55-59, 60-64, 65-69, 70-74, 75-79, 80-84, and ≥ 85 years), sex, month of test, and Trust area. Abbreviations: CI, confidence interval.

**
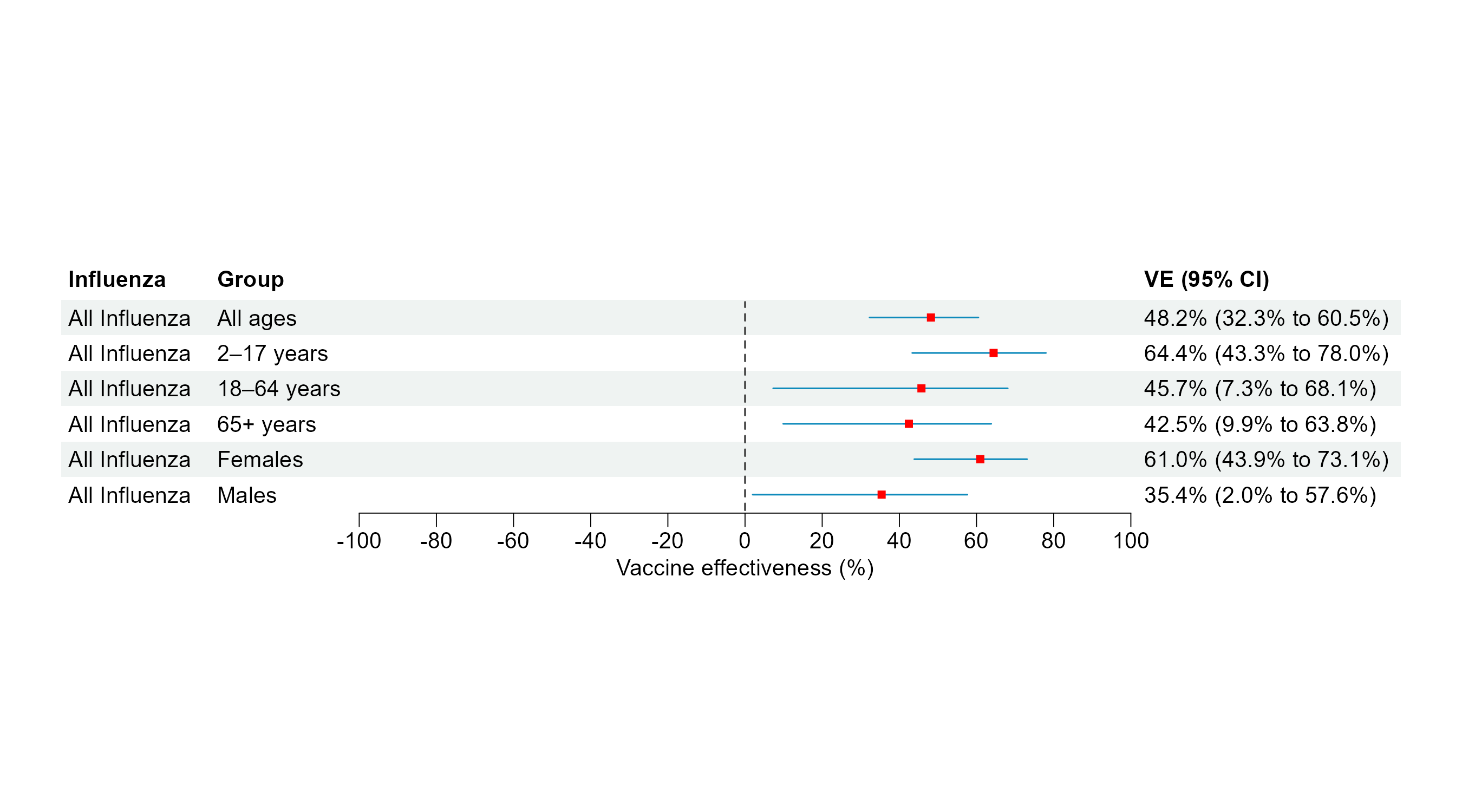
**

**Figure S2.** Vaccine effectiveness estimates including controls who tested positive for SARS-CoV-2. VE adjusted for age group (2–9, 10-17, 18-24, 25-29, 30-34, 35-39, 40-44, 45-49, 50-54, 55-59, 60-64, 65-69, 70-74, 75-79, 80-84, and ≥ 85 years), sex, month of test, and HSC Trust. Abbreviations: CI, confidence interval.


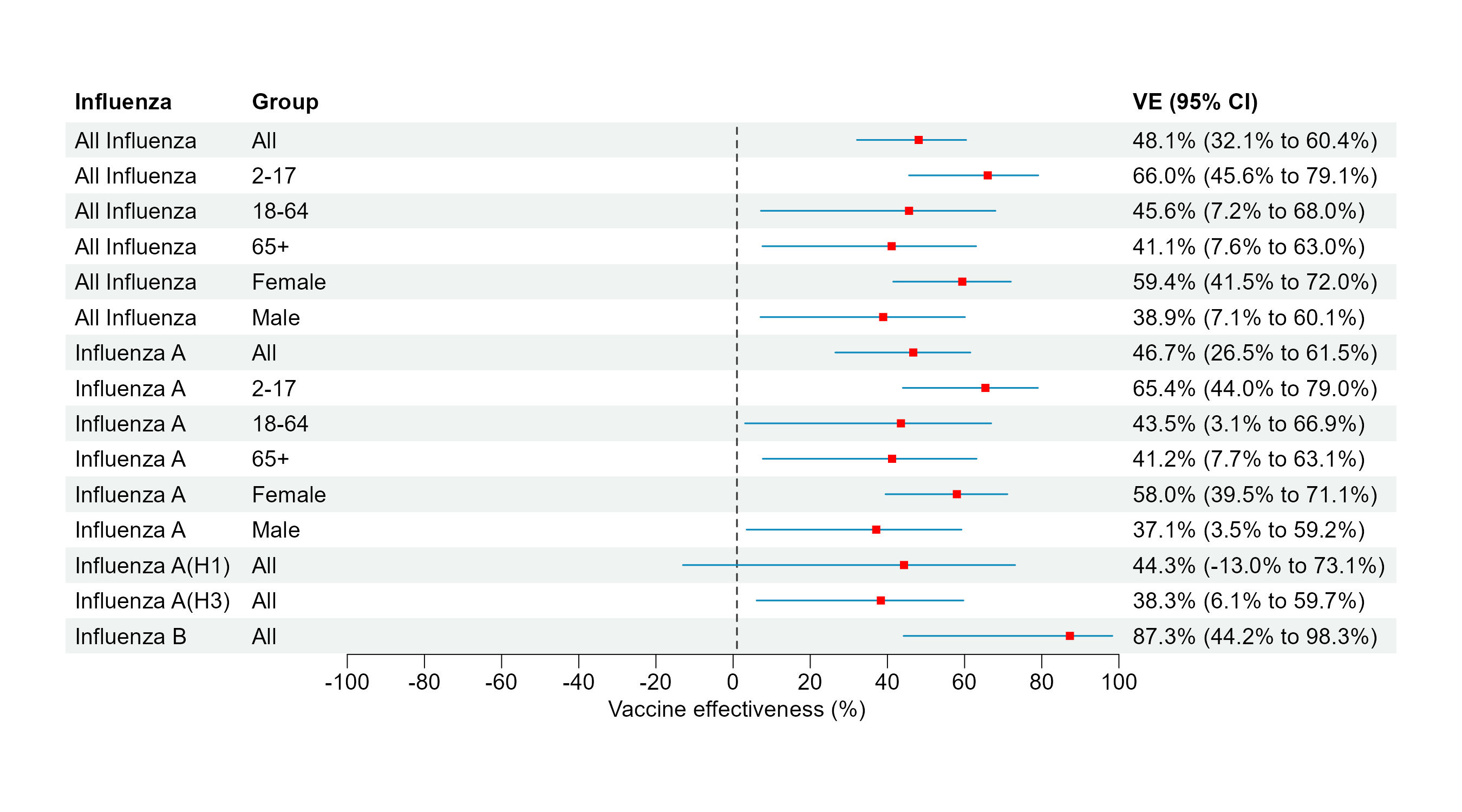


**Figure S3.** Vaccine effectiveness estimates against all influenza and by influenza type/subtype, age group, and sex. All odds ratios were adjusted for sex, month of test, HSC Trust, and age, parametrised as a restricted cubic spline, with k-knots determined separately for different sub-analyses. Abbreviations: CI, confidence interval.


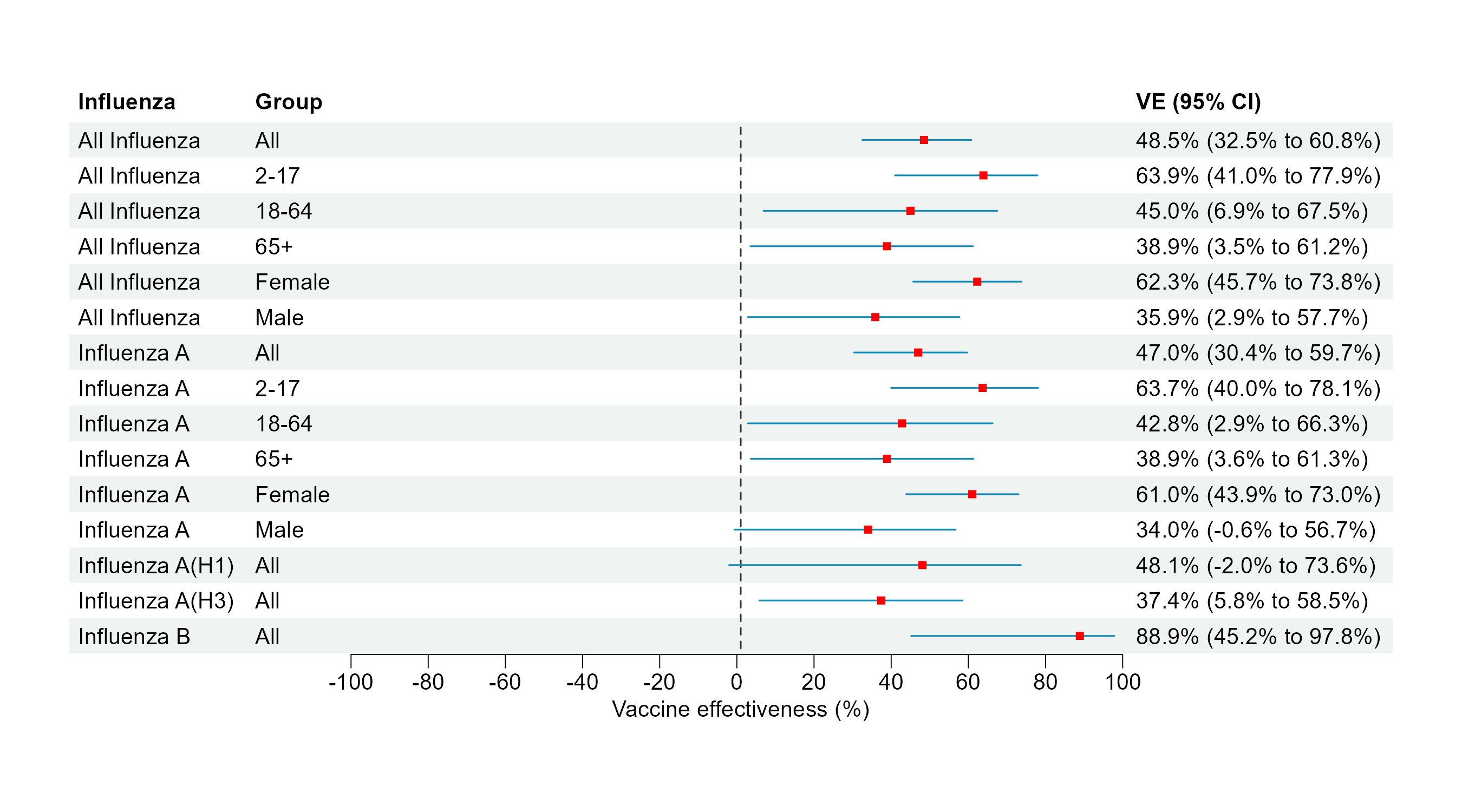


**Figure S4.** Vaccine effectiveness estimates against influenza B, A(H1), and A(H3) based on Firth’s penalised logistic regression models. VE adjusted for age group (2–9, 10-17, 18-24, 25-29, 30-34, 35-39, 40-44, 45-49, 50-54, 55-59, 60-64, 65-69, 70-74, 75-79, 80-84, and ≥ 85 years), sex, month of test, and HSC Trust. Error bars represent 95% confidence intervals (CI).


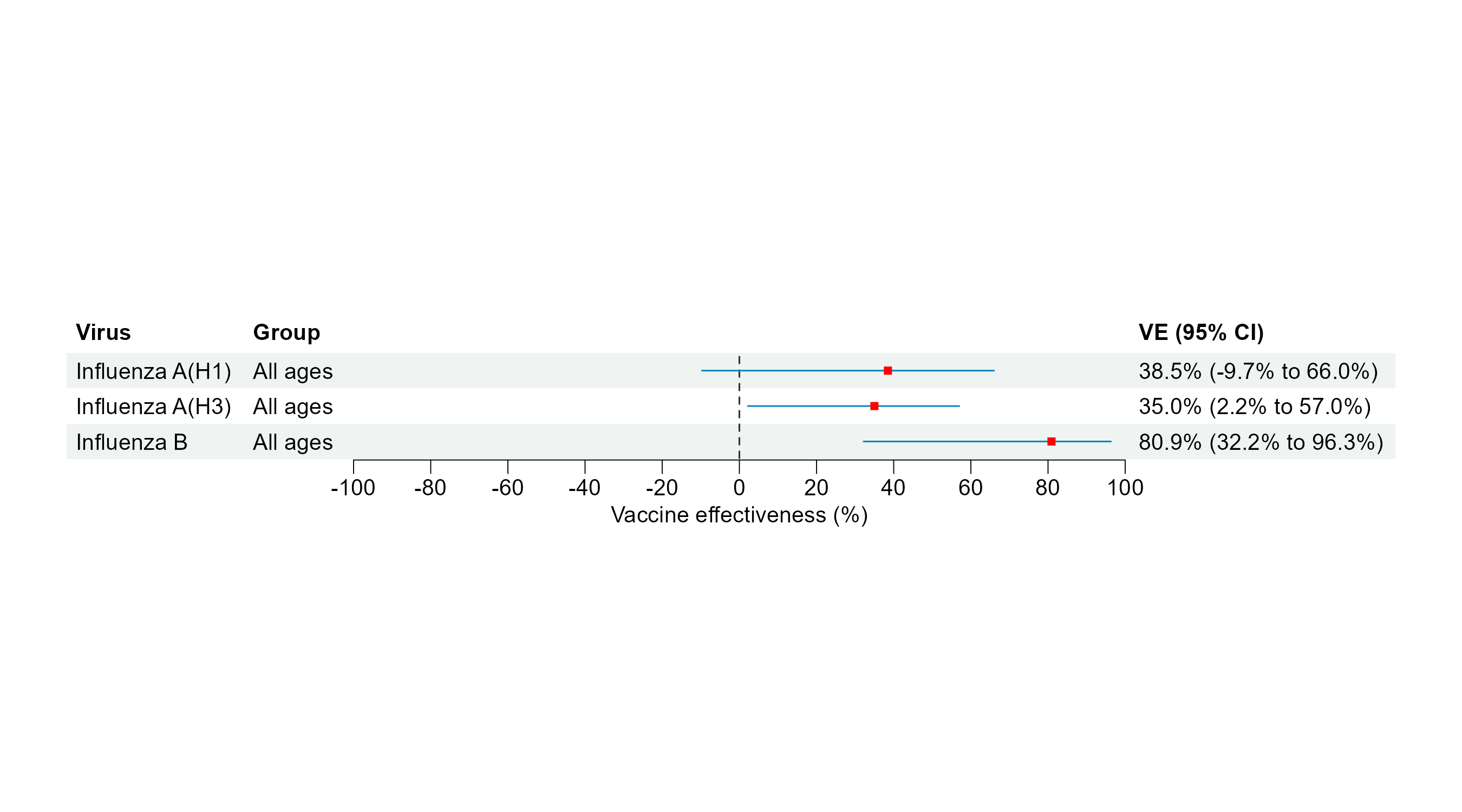

Supplement: Supplementary file 1 — Figure S1: Vaccine effectiveness estimates including partially vaccinated individuals (sample collected 7–14 days post vaccination). VE adjusted for age group (2–9, 10–17, 18–24, 25–29, 30–34, 35–39, 40–44, 45–49, 50–54, 55–59, 60–64, 65–69, 70–74, 75–79, 80–84 and ≥ 85 years), sex, month of test and trust area. CI, confidence interval. Figure S2: Vaccine effectiveness estimates including controls who tested positive for SARS‐CoV‐2. VE adjusted for age group (2–9, 10–17, 18–24, 25–29, 30–34, 35–39, 40–44, 45–49, 50–54, 55–59, 60–64, 65–69, 70–74, 75–79, 80–84 and ≥ 85 years), sex, month of test and HSC Trust. CI, confidence interval. Figure S3: Vaccine effectiveness estimates against all influenza and by influenza type/subtype, age group and sex. All odds ratios were adjusted for sex, month of test, HSC Trust and age, parametrised as a restricted cubic spline, with k‐knots determined separately for different sub‐analyses. CI, confidence interval. Figure S4: Vaccine effectiveness estimates against influenza B, A(H1) and A(H3) based on Firth's penalised logistic regression models. VE adjusted for age group (2–9, 10–17, 18–24, 25–29, 30–34, 35–39, 40–44, 45–49, 50–54, 55–59, 60–64, 65–69, 70–74, 75–79, 80–84 and ≥ 85 years), sex, month of test and HSC Trust. Error bars represent 95% confidence intervals (CI). [file IRV-19-e70149-s001.docx]
